# Hibernation and hemostasis

**DOI:** 10.3389/fphys.2023.1207003

**Published:** 2023-06-26

**Authors:** Edwin L. De Vrij, Hjalmar R. Bouma, Robert H. Henning, Scott T. Cooper

**Affiliations:** ^1^ Department of Plastic Surgery, University Medical Center Groningen, University of Groningen, Groningen, Netherlands; ^2^ Department of Clinical Pharmacy and Pharmacology, University Medical Center Groningen, Groningen, Netherlands; ^3^ Department of Internal Medicine, University Medical Center Groningen, University of Groningen, Groningen, Netherlands; ^4^ Biology Department, University of Wisconsin-La Crosse, La Crosse, WI, United States

**Keywords:** hibernation, torpor, hemostasis, platelet, coagulation, metabolism, hypothermia

## Abstract

Hibernating mammals have developed many physiological adaptations to accommodate their decreased metabolism, body temperature, heart rate and prolonged immobility without suffering organ injury. During hibernation, the animals must suppress blood clotting to survive prolonged periods of immobility and decreased blood flow that could otherwise lead to the formation of potentially lethal clots. Conversely, upon arousal hibernators must be able to quickly restore normal clotting activity to avoid bleeding. Studies in multiple species of hibernating mammals have shown reversible decreases in circulating platelets, cells involved in hemostasis, as well as in protein coagulation factors during torpor. Hibernator platelets themselves also have adaptations that allow them to survive in the cold, while those from non-hibernating mammals undergo lesions during cold exposure that lead to their rapid clearance from circulation when re-transfused. While platelets lack a nucleus with DNA, they contain RNA and other organelles including mitochondria, in which metabolic adaptations may play a role in hibernator’s platelet resistance to cold induced lesions. Finally, the breakdown of clots, fibrinolysis, is accelerated during torpor. Collectively, these reversible physiological and metabolic adaptations allow hibernating mammals to survive low blood flow, low body temperature, and immobility without the formation of clots during torpor, yet have normal hemostasis when not hibernating. In this review we summarize blood clotting changes and the underlying mechanisms in multiple species of hibernating mammals. We also discuss possible medical applications to improve cold preservation of platelets and antithrombotic therapy.

## Overview of hemostasis

**FIGURE 1:**
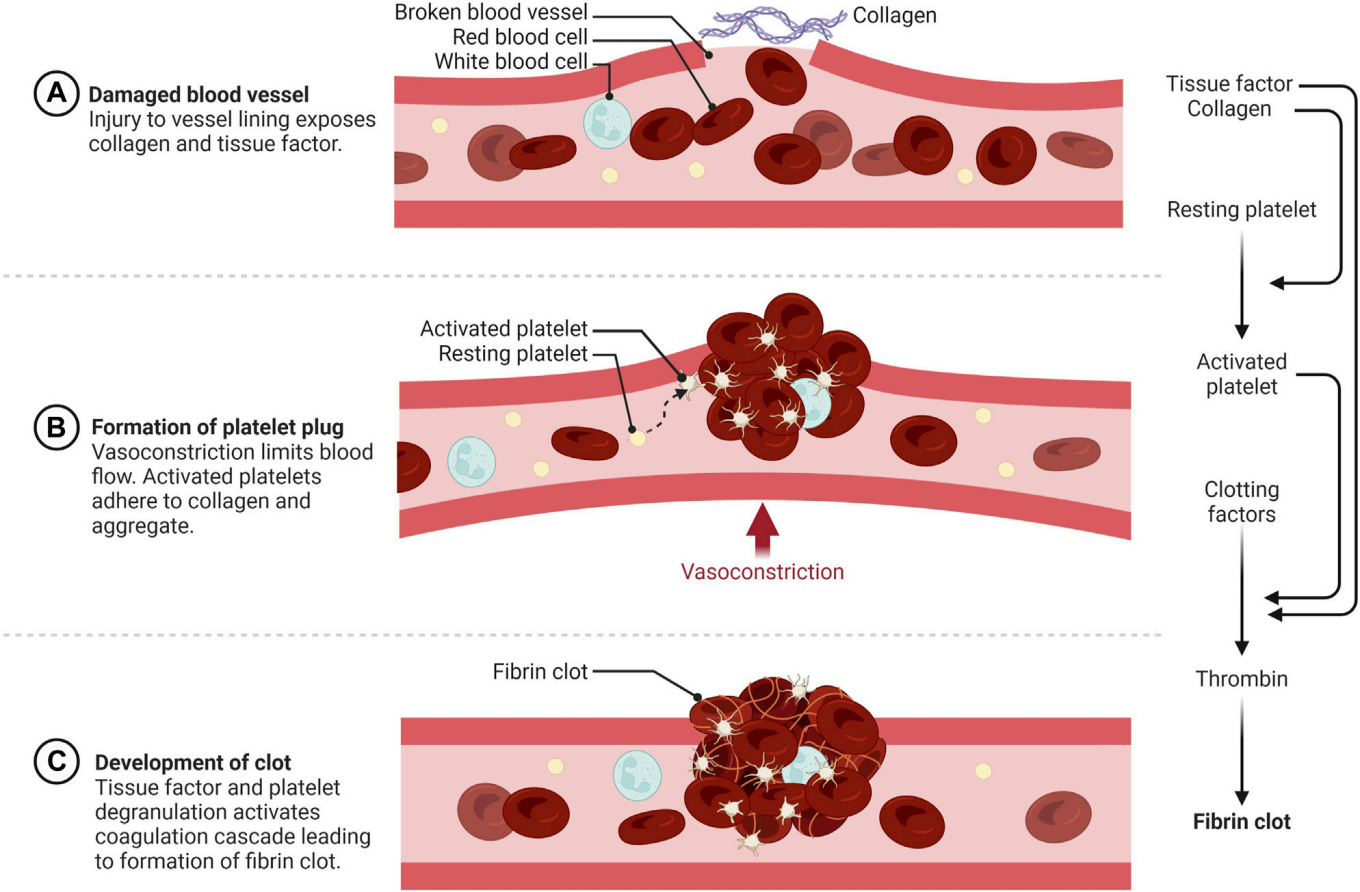
Schematic overview of hemostasis. **(A)** Primary hemostasis is initiated by vessel wall injury exposing the extracellular matrix (ECM) to blood. **(B)** Activated and degranulated platelets initiate platelet and white blood cell recruitment to form a platelet plug. This accelerates platelet aggregation, vasoconstriction, wound regeneration, and activation of secondary hemostasis. **(C)** Secondary hemostasis occurs via activation of the plasma coagulation system due to exposure of plasma zymogens to tissue factor (TF) and platelet activation products, forming a fibrin rich network that traps red and white blood cells and further strengthens the hemostatic plug https://biorender.com/.

Blood plays an essential role in vertebrates by circulating oxygen and nutrients and removing wastes from all tissues. This requires an extensive network of vessels that contain fluid blood, yet can form solid clots if vessels are damaged to prevent the organism from bleeding to death. Spontaneous blot clot formation is counterbalanced by anticoagulant mechanisms that can become overwhelmed at the site of a wound leading to hemostasis. The word hemostasis is derived from the Greek αίμα/hema (=blood) and στάσις/stasis (=halt), literally the stopping of blood. Hemostasis is generally divided into three phases, primary hemostasis involving anucleated cells called platelets, secondary hemostasis triggered by a cascade of serine proteases to form a fibrin clot, and fibrinolysis, which breaks down the clot (Figure 1). There are multiple ways to activate hemostasis including endothelial cell injury, clotting factors or platelet alterations, and abnormal blood flow. During torpor, heart rate and blood flow are reduced to levels that would lead to the formation of potentially lethal blood clots in most non-hibernating mammals. Hypothermia also affects both procoagulant and anticoagulant aspects of hemostasis. Hibernating mammals thus need multiple adaptations to reversibly suppress blood clotting during torpor, which will be discussed in this review.

## Hibernating mammalian models

Hibernators encounter several risk factors for thrombosis including low body temperature, stasis of blood, hypercoagulability, and endothelial activation. During torpor a ground squirrel’s body temperature drops from 35°C–38°C to 4°C–8°C (Lechler and Penick, 1963; Reddick et al., 1973), the heart rate is reduced from its normal 200–300 to three to five beats/min (Zatzman, 1984), and respiration is reduced from 100 to 200 to four to six breaths/min (McArthur and Milsom, 1991). As expected, blood pressure also drops from 140/100 mmHg to 60/30 mmHg, with values as low as 10 mmHg reported (Lyman and O’brien 1960). Thus hibernation entails periods of prolonged immobility (Carey et al., 2003; Utz et al., 2009) with low blood flow (stasis) in veins and atria (Horwitz et al., 2013), increased blood viscosity (Kirkebo, 1968; Halikas and Bowers, 1973; Arinell et al., 2018), cycles of hypoxia-reoxygenation, and cooling-rewarming with signs of endothelial activation (Carey et al., 2003; Talaei et al., 2012). Additionally, at entrance of the hibernation season, hibernators are generally grossly overweight (Martin, 2008). Remarkably, despite this multitude of risk factors for thromboembolism, hibernators do not demonstrate signs of organ damage due to thromboembolic complications during hibernation or upon arousal in spring (Cooper et al., 2012; Bonis et al., 2019).

**FIGURE 2:**
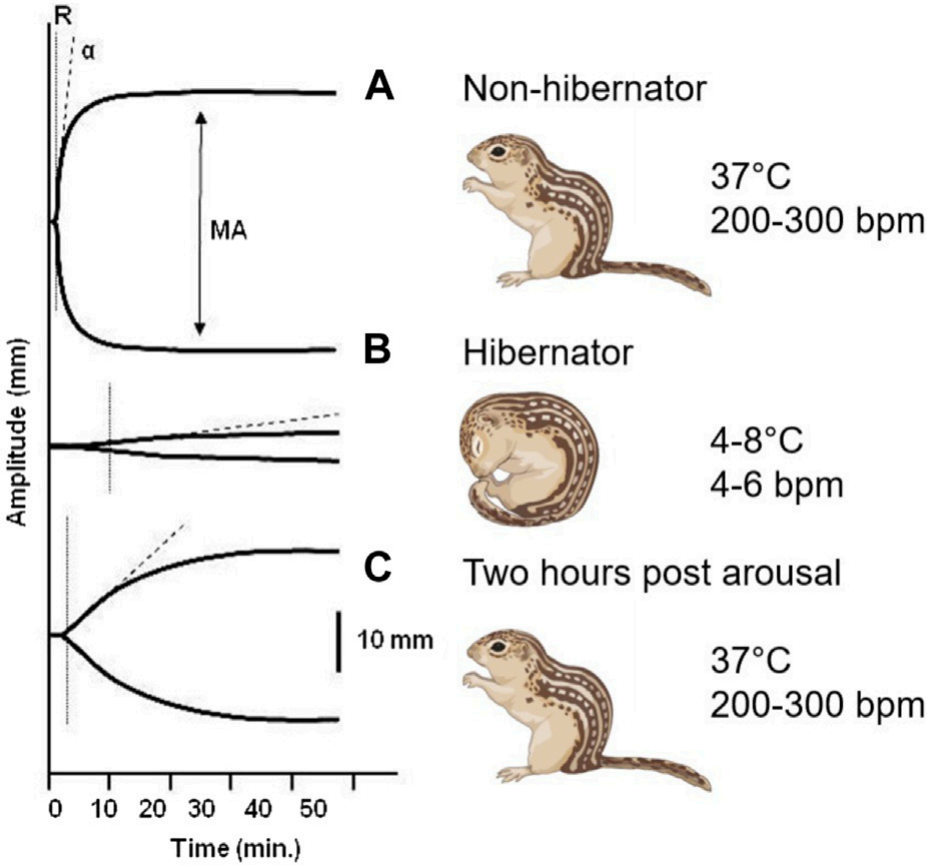
Thromboelastogram of 13-lined ground squirrel blood collected at different hibernation phases. **(A)** During summer, the rate of formation (R) and strength of the clot (MA, maximum amplitude) reflect fully functional primary and secondary hemostasis. **(B)** The time needed to form a clot increases and the strength of the clot dramatically decreases during the torpor phase of hibernation. **(C)** Within 2 hours of arousal both primary and secondary hemostasis are restored to functional levels (Cooper et al., 2012).

A reversible decrease in hemostasis has been demonstrated during torpor in diverse hibernating animals. In small mammalian deep hibernators body temperature can drop to 2°C–4°C as found in ground squirrels (Svihla et al., 1951; Lechler and Penick, 1963; Reddick et al., 1973; Pivorun and Sinnamon, 1981; Bouma et al., 2010; Cooper et al., 2012; Hu et al., 2017; Öztop et al., 2019), hamsters (Denyes and Carter, 1961; Deveci et al., 2001; de Vrij et al., 2014), hedgehogs (Biorck et al., 1962; De Wit et al., 1985), and bats (Rashid et al., 2016). In contrast, large mammals like bears show more moderate body temperatures during hibernation by only dropping from 37°C to 32°C coupled with prolonged immobility (Fröbert et al., 2010; Welinder et al., 2016; Iles et al., 2017; Arinell et al., 2018; Thienel et al., 2023). Decreased hemostasis in hibernators may partly be explained by the decrease in body temperature, as forced hypothermia also lowers platelet counts in hamsters, and even suppresses hemostasis in non-hibernating species like rats and mice (de Vrij et al., 2014). Finally, ectothermic vertebrates also reduce hemostasis during hibernation as seen in turtles (Barone and Jacques, 1975) and frogs (Ahmad et al., 1979). Given that a wide range of hibernating species suppress their hemostasis during torpor through multiple mechanisms, it is likely that this represents a vital physiological adaptation to multiple risk factors during torpor. The rapid restoration of hemostasis after arousal indicates that being able to form clots soon after arousal is also imperative. Changes in blot clot formation throughout hibernation is illustrated graphically by thromboelastograms where whole blood is treated with an activator of coagulation and the speed and strength of clot formation is measured (Figure 2). Because both primary and secondary hemostasis are regulated by proteins in circulation and platelets which lack a nucleus, the adaptations to rapid changes in temperature and blood flow must be inherent in these proteins and cells, and not rely on new synthesis as seen in other organs.

## Effect of hypothermia on hemostasis

While clot formation may prevent bleeding after tissue damage and initiate wound healing, inadvertent generation of a thrombus can be detrimental for any organism. Virchow’s Triad was first described in 1856 and identified venous stasis, vascular injury, and hypercoagulability as risk factors for thrombosis. The ensuing disseminated intravascular coagulation (DIC) of hypothermic patients may result in consumption of clotting factors, ischemia, and necrosis of organs and eventually result in death (Duguid et al., 1961; Stine, 1977; Mahajan et al., 1981). Besides provoking prothrombotic effects, decreased body temperature slows the enzymatic reactions in secondary hemostasis (Rohrer and Natale, 1992; DeLong et al., 2017), prolonging bleeding time in cold skin, and diminishing thromboxane A release from platelets (Valeri et al., 1995; Michelson et al., 1999). Consequently, hypothermia is associated with a secondary hypocoagulation state with prolonged time to clot formation in tests such as PT and APTT when temperatures drop below 35°C (Rohrer and Natale, 1992; Polderman, 2009). Low temperature *in vivo* increases activation, aggregation, and reversible storage of platelets in the liver or spleen (Van Poucke et al., 2014). Low temperatures of the extremities have been implicated to ‘prime’ platelets for activation at these sites most susceptible to bleeding throughout evolutionary history, which also leads to increased clearance of these platelets from circulation (Hoffmeister et al., 2003). Furthermore, both accidental and therapeutic hypothermia are associated with a reduction in platelet count known as thrombocytopenia (Vella et al., 1988; Chan and Beard, 1993; Mikhailidis and Barradas, 1993; Mallet, 2002; Morrell et al., 2008; Jacobs et al., 2013; Wang et al., 2015). Whether this thrombocytopenia in patients can be reversed quickly by rewarming—or whether thrombocytopenia recovers due to *de novo* production and release of platelets from the bone marrow—is still not clear.

*Ex vivo* cooling of platelets from non-hibernating mammals induces platelet shape changes similar to activation of platelets (White and Krivit, 1967; Winokur and Hartwig, 1995; Peerschke et al., 2008; Egidi et al., 2010), while low temperature also increases degranulation of activated platelets and plasma levels of activation products of platelets (Hoffmeister, 2011; Van Poucke et al., 2014). Cooled platelets demonstrate an increased tendency to aggregate (Xavier et al., 2007) and are rapidly cleared from circulation after transfusion (Becker et al., 1973; Berger et al., 1998; Van Poucke et al., 2014). Currently, human platelets are stored at 22°C–24°C room temperature before transfusion which increases risk of bacterial contamination and thus limits shelf-life to only 5–7 days, compared to 40 days for cold stored red blood cells (D'Alessandro et al., 2010). However, even if low body temperature leads to a 10-fold decrease in enzymatic activity and slowing down of clot formation, the prolonged time in torpor would be sufficient for clots to form during torpor. For example, to produce serum in the lab, blood is collected in the absence of an anticoagulant and stored in a refrigerator where it will still form a fibrin clot at 4°C if given enough time. Further unraveling the temperature effects on hemostasis may yield improved understanding of its role in hypothermic patients and in hibernating mammals and potentially disclose new pathways for drug development focused on novel antithrombotic strategies.

**FIGURE 3:**
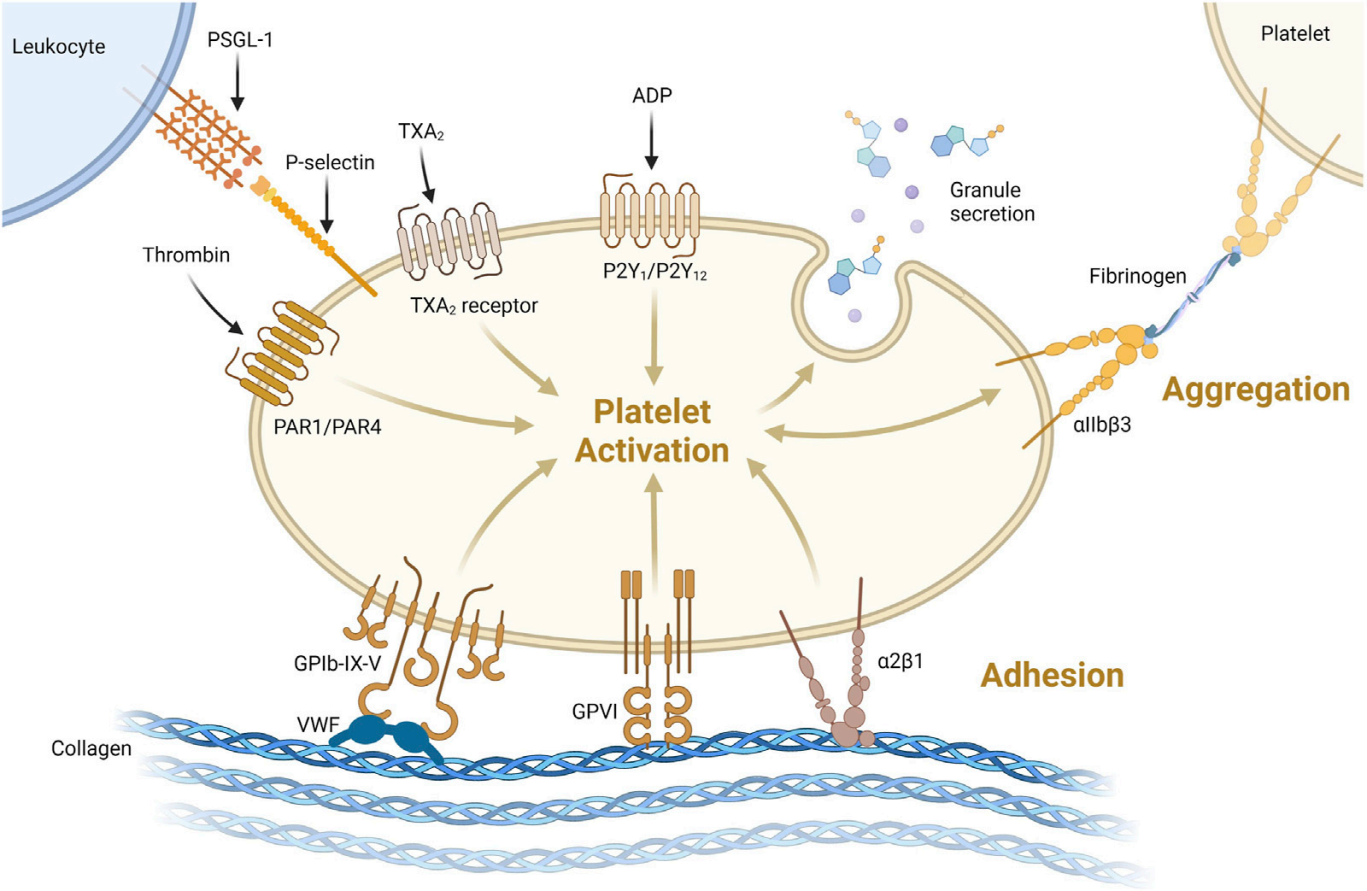
Platelets can be activated through one or more pathways. Illustrated here are activation via thrombin binding PAR1/PAR4, thromboxane 2 (TXA_2_) binding TXA_2_receptor or ADP binding P_2_Y_1_/P_2_Y_12_ receptor. Platelets can be activated and bind collagen via VWF binding GPIb-IX-V or directly via GPVI or α2β1 receptors binding collagen. Activation leads to degranulation, secreting its granule content. Platelet-platelet aggregation is supported via αIIbβ3 binding via fibrinogen as shown on the top right. Platelet-leukocyte interaction acts via P-selectin binding to P-selectin glycoprotein 1 (PSGL-1, top left) https://biorender.com/.

Primary hemostasis involves adherence of circulating platelets to damaged endothelium or subendothelial collagen and aggregation between platelets (Figure 1A). Platelets, which bud off from large multinuclear bone marrow cells called megakaryocytes, are 2–5 µm anucleated blood cells that play a major role in hemostasis, inflammation, bacterial defense and wound regeneration and are even involved in cancer metastasis (Semple et al., 2011; Ware et al., 2013; Leblanc and Peyruchaud, 2016; Fernandez-Moure et al., 2017). Platelets are activated by many molecules including Von Willebrand Factor (VWF), collagen, thrombin, adenosine diphosphate (ADP) and adrenaline (Nieswandt et al., 2011; Versteeg et al., 2013). Activated platelets express membrane proteins including glycoprotein Ib-IX-V (GPIb-IX-V) and P-selectin that bind to activated endothelium or subendothelial collagen through VWF (Nieswandt et al., 2011) (Figure 3). Platelets degranulate upon activation, releasing molecules that stimulate further platelet activation, the coagulation cascade, inflammation, tissue regeneration and bacterial killing (Semple et al., 2011; Fernandez-Moure et al., 2017). More platelets are then recruited to the site of injury (Figure 1B). Platelets adhere to the subendothelial extracellular matrix (ECM) and forma hemostatic plug by aggregating with each other.

### Primary hemostasis adaptations during torpor

**FIGURE 4:**
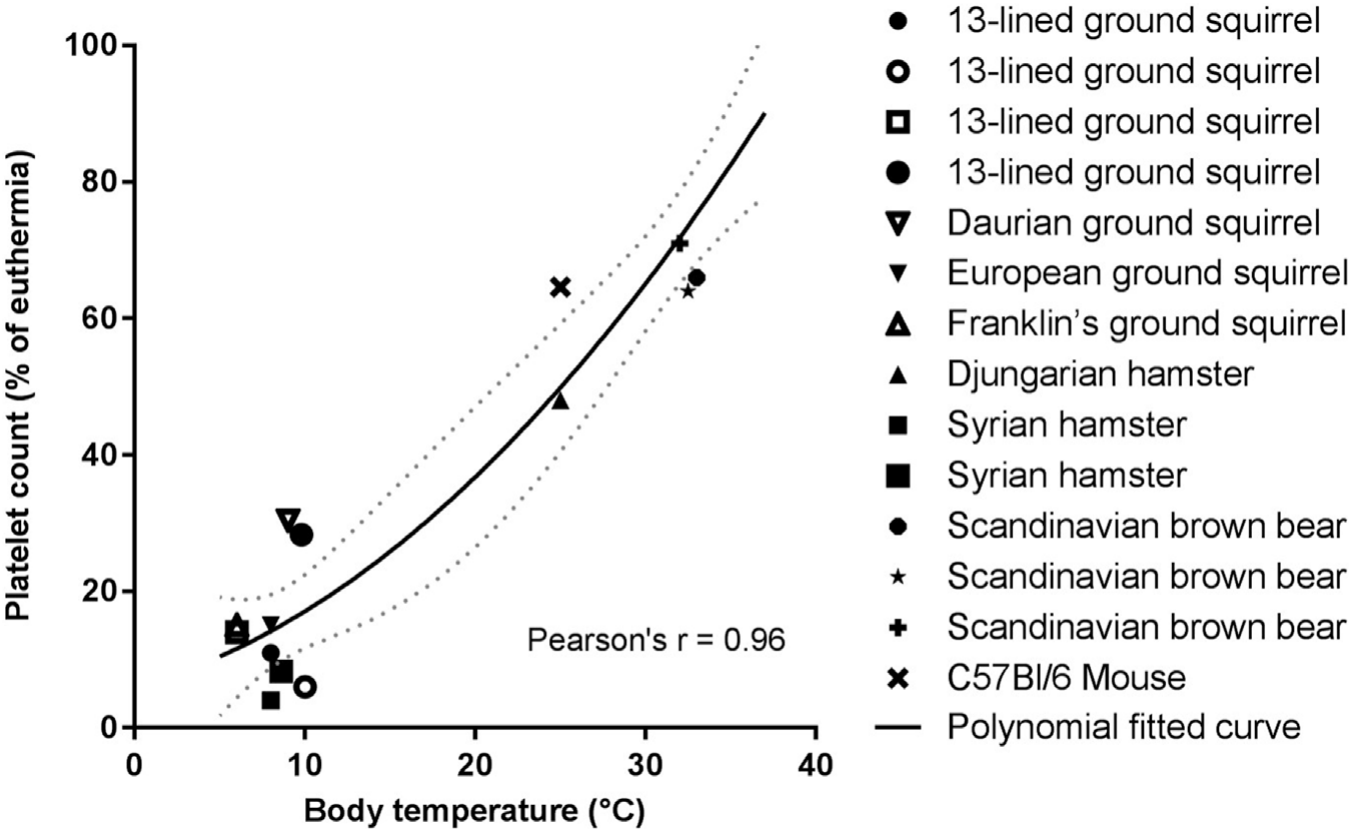
Temperature association with platelet count reduction is consistent for all hibernating mammals studied so far. Platelet count in torpor as percentage of euthermia platelet count was calculated from values of studies summarized in Table 1. If a study did not report euthermic platelet count, literature data were used. Fitted polynomial quadratic curve (black line) with 95% confidence interval (dotted gray line). Pearson’s r = 0.96, *p* < 0.05 (Lechler and Penick, 1963; Reddick et al., 1973; Pivorun and Sinnamon, 1981; Bouma et al., 2010; Fröbert et al., 2010; Cooper et al., 2012; de Vrij et al., 2014; Welinder et al., 2016; Cooper et al., 2017; Hu et al., 2017; Arinell et al., 2018; de Vrij et al., 2021).

Circulating platelet levels decrease about 10-fold in multiple hibernating species during torpor, with a return to normal levels as the animals rewarm during arousals (Figure 4). These dynamics could decrease the risk of a large intravascular clot forming during torpor, while restoring normal clotting activity upon arousal. The thrombocytopenia is temperature dependent and can be induced in hamsters, rats, and mice with forced chilling (de Vrij et al., 2014). Cold acclimation of rats and hamsters also reduced circulating platelet levels even though the animal’s body temperatures did not decrease (Deveci et al., 2001). The rapid return of platelets in circulation after arousal, in the absence of large numbers of circulating immature platelets, suggests that hibernator platelets are stored during the cold and released into circulation during rewarming. Removal of the spleens of Syrian hamsters and 13-lined ground squirrels before or during torpor did not affect the platelet storage and release indicating that spleen is not required for storage as originally proposed (Reddick et al., 1973; Cooper et al., 2012; Cooper et al., 2016; de Vrij et al., 2021). Rather, platelet storage during torpor occurs in the sinusoids of the liver, consistent with this organ being responsible for storing platelets (Cooper et al., 2017; de Vrij et al., 2021). The mechanism of hepatic storage of platelets during torpor is currently unknown, potentially platelet margination may underlie the reversible storage and release in liver (de Vrij et al., 2021). Although platelets cluster together in liver sinusoids during torpor, they do not form irreversible thrombi. Several adaptations may prevent platelet activation during their slumber in cold liver sinusoids. Platelet GpIbα receptors can bind to VWF, but the plasma concentration of VWF decreases during torpor in bears, ground squirrels and hamsters, with a selective loss of the thrombogenic high molecular weight multimers (Cooper et al., 2016; Friedrich et al., 2017). Additionally, platelets from torpid ground squirrels bind less VWF and collagen, consistent with decreased GpIbα activity on their surface. Upon arousal, VWF levels return to normal rapidly, possibly by release of stores in platelets or endothelial cells (Cooper et al., 2016). The combination of decreased platelet and VWF levels along with decreased binding of platelets to VWF would all help to suppress activation of primary hemostasis during torpor-induced stasis.

**TABLE 1:** Primary hemostasis in hibernation.

Measurement	Euthermia	Torpor	Arousal	Species	References
**Whole-blood clotting time**					
(sec)	210 ± 76	315 ± 66*		13-lined ground squirrel	Lechler and Penick (1963)
(min)	2.2 ± 0.3	48.0 ± 5.4*	11.5 ± 1.5*^,#^	Franklin’s ground squirrel	Pivorun and Sinnamon (1981)
(min)	4	11	5	Hedgehog	Biorck et al. (1962)
(sec)	81 ± 12	217 ± 30	164 ± 33	American black bear	Iles et al. (2017)
**Thromboelastography**					
R (min)	1.6 ± 0.4	16.2 ± 12.3*	4.7 ± 1.2*^,#^	13-lined ground squirrel	Cooper et al. (2012)
Alpha (°)	58.8 ± 11.3	6.6 ± 6.2*	17.1 ± 7.4*^,#^	13-lined ground squirrel	Cooper et al. (2012)
Maximum amplitude (mm)	47.2 ± 5.2	6.0 ± 6.8*	17.2 ± 10.4*^,#^	13-lined ground squirrel	Cooper et al. (2012)
G (dynes/cm^2^)	4.6 ± 1.0	0.3 ± 0.4*	1.1 ± 0.8*^,#^	13-lined ground squirrel	Cooper et al. (2012)
**Platelet aggregation** (arbitrary units)					
ADP	70.0 ± 26.6	29.2 ± 8*		Scandinavian brown bear	Arinell et al. (2018)
	66 ± 23	33 ± 10*		Scandinavian brown bear	Arinell et al. (2018)
Arachidonic acid	73 ± 16	28 ± 9*		Scandinavian brown bear	Arinell et al. (2018)
	68 ± 20	33 ± 10*		Scandinavian brown bear	Arinell et al. (2018)
Collagen	68.3 ± 17	30.7 ± 10		Scandinavian brown bear	Arinell et al. (2018)
	63 ± 22	30 ± 7*		Scandinavian brown bear	Arinell et al. (2018)
TRAP	18.5 ± 10.0	9.2 ± 6.9*		Scandinavian brown bear	Arinell et al. (2018)
PAR-4	22.5 ± 7.1	12.7 ± 7.1*		Scandinavian brown bear	Arinell et al. (2018)
**Platelet count**					
(x 10^9^/L)	445.2 ± 123.4 36.3°C	47.9 ± 22.3* 7.9°C		13-lined ground squirrel	Lechler and Penick (1963)
	303.6 ± 10.6 37°C	45 ± 3.4* 6°C	232.4 ± 19.7*^,#^ 37°C	Franklin’s ground squirrel	Pivorun and Sinnamon (1981)
	375.3 ± 40.8 37°C	114.2 ± 36.0* 9°C	217.0 ± 35.9*^,#^ 37°C	Daurian ground squirrel	Hu et al. (2017)
	293 ± 81 36°C	44 ± 30.9* 8°C	194 ± 5*^,#^ 35°C	European ground squirrel	Bouma et al. (2010)
		23.3 ± 1.3* 9.8°C	410.9 ± 59.2 36°C	13-lined ground squirrel	Cooper et al. (2012)
	394 ± 157	55 ± 30*		13-lined ground squirrel	Reddick et al. (1973)
	451 ± 87 35.5°C	128 ± 81 9.8°C	498 ± 144 36.4°C	13-lined ground squirrel	Cooper et al. (2017)
	797 ± 124 35°C	381 ± 239* 25°C	739 ± 253^#^ 35°C	Djungarian hamster	de Vrij et al. (2014)
	198 ± 6 35°C	8 * 8°C	187^#^ 35°C	Syrian hamster	de Vrij et al. (2014)
	430 ± 82 35.7°C	36 ± 17* 8.2°C	468 ± 100 35.4°C	Syrian hamster	de Vrij et al. (2021)
	207 ± 24			Scandinavian brown bear	Fröbert et al. (2010)
	262 ± 61 40°C	174 ± 51* 33°C	262 ± 61	Scandinavian brown bear	Arinell et al. (2018)
	229 ± 39	146 ± 47*		Scandinavian brown bear	Arinell et al. (2018)
	228 ± 36 37°C	149 ± 43* 32°C		Scandinavian brown bear	Welinder et al. (2016)
(%EU)	100% ± 31% (n = 19)	65% ± 29% * (n = 12)	111% ± 26% (n = 13)	Daily torpor C57Bl/6 Mouse	(De Vrij et al. unpublished)
	34°C	26°C	33°C		
(10^2^/mm^3^ Mean ± SE)	5.6 ± 0.6		6.0 ± 1.1	Common yellow bat	Rashid et al. (2016)
(10^2^/mm^3^ Mean ± SE)	7.5 ± 1.0		7.4 ± 1.2	Common pipistrelle bat	(Rashid et al., 2016)
**P-selectin expressing platelets** (%)	8 ± 7	0	7 ± 6	Syrian hamster	de Vrij et al. (2014)
	3 ± 1	11 ± 7	10 ± 3	Syrian hamster	de Vrij et al. (2021)
**Platelets activated by ADP** (%)	16 ± 14	16 ± 6	29 ± 6	Syrian hamster	de Vrij et al. (2014)
	22 ± 8	16 ± 2	47 ± 11*^,#^	Syrian hamster	de Vrij et al. (2021)
**VWF** (% relative to human plasma)	24.9 ± 3.7	2.4 ± 0.01*		13-lined ground squirrel	Cooper et al. (2016)
(%EU)	100% ± 10.5% (n = 10)	7.3% ± 3.2%* (n = 4)	8.4% ± 11.5%* (n = 4)	Syrian hamster	(De Vrij et al. unpublished)
(IU/mL)	1.7 ± 0.2	1.3 ± 0.2*		Scandinavian brown bear	Welinder et al. (2016)
**VWF:collagen binding activity** (%EU)	100% ± 10% (n = 7)	35% ± 15% (n = 4)	25% ± 33%* (n = 4)	Syrian hamster	(De Vrij et al. unpublished)

Shape change is another prominent feature of cooled and hibernating platelets. Under normal conditions, platelets have a circumferential microtubule ring that keeps them in a disc conformation. In non-hibernating mammals, chilling platelets leads to depolymerization of their microtubules and the platelets become spherical and do not repolymerize upon warming (White and Krivit, 1967; Winokur and Hartwig, 1995). In contrast, ground squirrels and Syrian hamster platelet microtubules form rods when chilled and can reconstitute the circumferential ring upon rewarming (Cooper et al., 2017; de Vrij et al., 2021). This allows ground squirrel platelets to retain their overall shape after chilling. It is unclear if this rod shape has any physiological or adaptive significance, but it may protect platelets from damage caused by cold storage.

Cold storage of human and mouse platelets damages platelets in several ways, collectively termed cold storage lesions. Sialic acid residues on surface glycoproteins are cleaved leading to the rapid clearance of chilled platelets from circulation by the liver (Hoffmeister et al., 2003; Hoffmeister, 2011). Cold storage also induces intrinsic mitochondrial activated apoptosis in human and mouse platelets (Van Der Wal et al., 2010; Lebois and Josefsson, 2016; Stolla et al., 2020). In contrast, ground squirrel platelets stored *in vitro* in the cold are not cleared rapidly upon re-transfusion (Cooper et al., 2012). Ground squirrel platelets stored in the cold show decreased desialylation of surface glycoproteins and decreased phagocytosis by hepatocytes. They also appear to be resistant to induction of apoptosis as measured by caspase activation and surface phosphatidylserine on platelet membranes (Splinter et al., 2023). These adaptations of hibernator platelets to cold storage are consistent with their storage and release after weeks of cold storage during torpor and likely add to the rapid restoration of a hibernator’s ability to form clots upon arousal.

Platelets are anucleate cells and their protein content is determined primarily by progenitor megakaryocytes in the bone marrow. Surprisingly, a bone marrow transcriptome of samples collected in the fall, winter, and summer from 13-lined ground squirrels revealed no significant differences in any platelet protein transcripts, including P-selectin, membrane receptors, and integrins (Cooper et al., 2016). The platelet proteome collected in the same species and at the same times of the year, also displayed no differences in integrins and signaling proteins, but did reveal significant seasonal differences in other proteins (Cooper et al., 2021). In the summer, platelets have increased heat shock proteins which could help with proper protein folding at warm temperatures. In the fall, as animals are going through short bouts of shallow torpor, pro-inflammatory and clotting proteins become less abundant in platelets. During torpor, more plasma-derived proteins such as albumin and lipoproteins are present in platelets which are known to inhibit apoptosis and thrombosis (Cooper et al., 2021). A very recent platelet proteome study on brown bears showed a 55-fold decrease in the heat shock protein HSP47 (SerpinH1) during hibernation (Thienel et al., 2023). This decrease correlated with a decline in DVT formation during prolonged immobilization, and was also demonstrated in humans, pigs, and knock-out mice. Interestingly, and in sharp contrast, a previous study of 13-lined ground squirrel platelet proteome documented unchanged HSP47 abundance across various phases of hibernation (Cooper et al., 2021). This marked difference in Hsp47 regulation could be due to bears being shallow hibernators and also of greater body mass compared to ground squirrel, so the immobilization puts bears at greater risk of DVT. Moreover, torpid bears have a modest drop in circulating platelets compared to deep hibernators (Figure 4), possibly warranting additional protection against DVT. It would be interesting to look more closely at the inhibition of HSP47 in deep hibernators other than 13 lined ground squirrel to appreciate its role in hibernation. In addition to the decrease in circulating platelet numbers, these proteomic changes could prevent unwanted activation of platelets during stasis or during their storage in liver. While there were no significant differences in the amounts of surface receptors or proteins in signaling pathways at the proteome level, it is possible that the activities of these pathways are altered. Alternatively, some of the proteins may be sequestered in the cytoplasm or secretory granules which could be resolved by immunohistochemical analysis. Studying the phosphoproteome and metabolome of platelets in torpor would help to resolve which pathways are affected.

**FIGURE 5:**
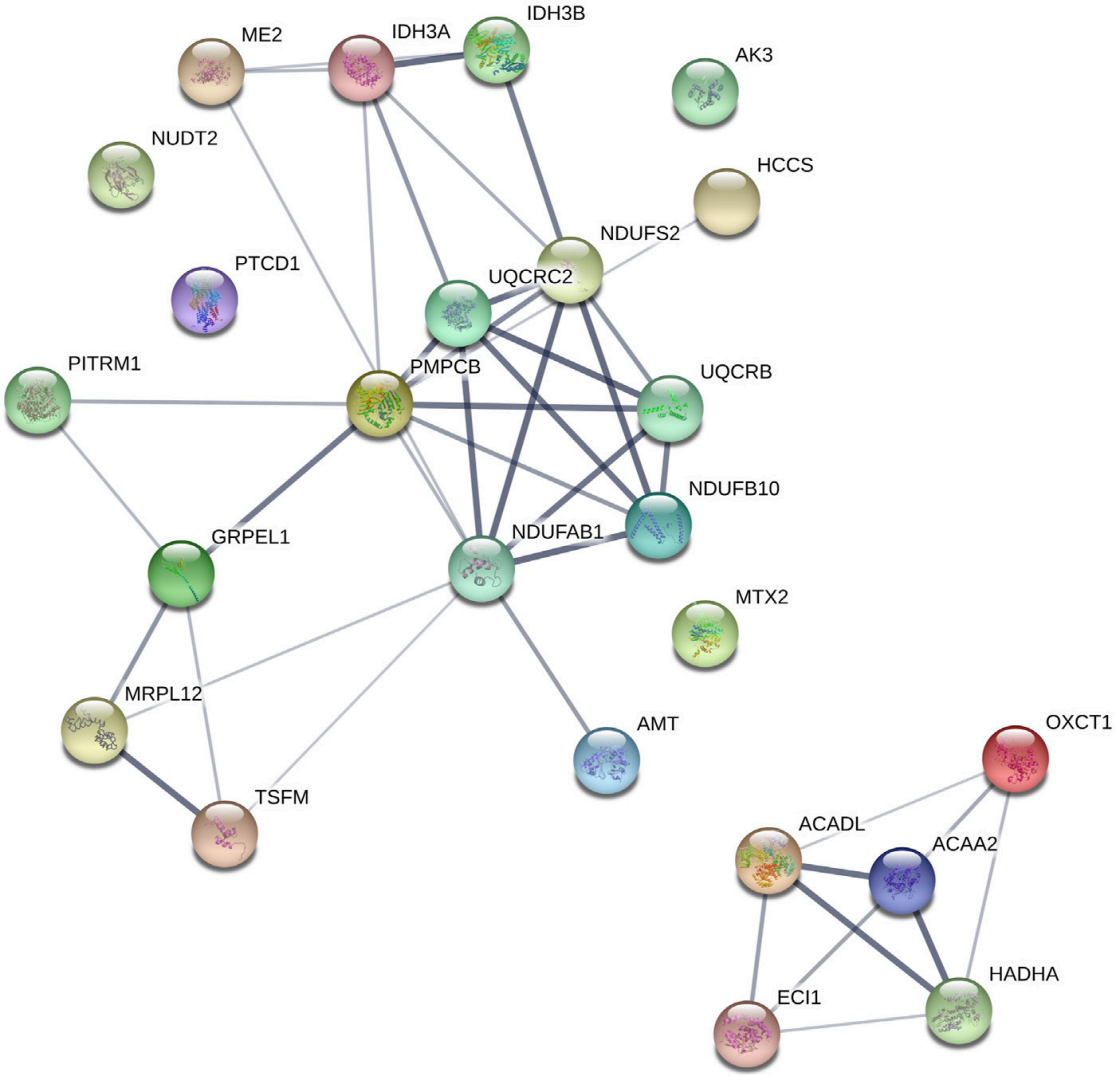
Mitochondrial proteins upregulated during winter clustered using STRING. OXCT1 (Succinyl-CoA-3-ketoacid coenzyme A transferase 1), ACADL (Long-chain specific acyl-CoA dehydrogenase), PMPCB (Mitochondrial-processing peptidase subunit beta), MTX2 (Metaxin-2); GRPEL1 (GrpE protein homolog 1), UQCRC2 (Cytochrome b-c1 complex subunit 2), NDUFB10 (NADH dehydrogenase [ubiquinone] 1 beta subcomplex subunit 10), AMT (Aminomethyltransferase), ACAA2 (3-ketoacyl-CoA thiolase), PTCD1 (Pentatricopeptide repeat-containing protein 1), IDH3 (AIsocitrate dehydrogenase [NAD] subunit alpha), ECI1 (Enoyl-CoA delta isomerase 1), TSFM (Elongation factor Ts), ME2 (NAD-dependent malic enzyme), HCCS (Cytochrome c-type heme lyase), MRPL12 (39S ribosomal protein L12), NDUFS2 (NADH dehydrogenase [ubiquinone] iron-sulfur protein 2), NUDT2 (Bis(5′-nucleosyl)-tetraphosphatase), HADHA (Trifunctional enzyme subunit alpha), IDH3B (Isocitrate dehydrogenase [NAD] subunit beta), PITRM1 (Presequence protease), AK3 (GTP-AMP phosphotransferase AK3), UQCRB (Cytochrome b-c1 complex subunit 7), NDUFAB1 (Acyl carrier protein).

In recent years, cultured cells from hibernators have demonstrated to hold specific adaptations that confer protection from cooling in a cell-autonomous way (Talaei et al., 2011; Ou et al., 2018; Hendriks et al., 2020). Mechanistically, these studies in hamster cell lines and ground squirrel pluripotent stem cells derived neurons indicate that adaptations in mitochondrial function, allowing cooled cells to maintain ATP production and lower reactive oxygen species production, constitute a key element of cell protection. Given that platelets contain mitochondria, we re-analyzed aforementioned ground squirrel platelet proteome data (Cooper et al., 2021) by separating out mitochondrial proteins. Analysis of the platelet proteins involved in metabolism revealed an increase in 79 proteins with clusters in lipoprotein metabolism, cholesterol esterification, and lipase activity during the winter. With respect to mitochondrial proteins, 24 were increased during the winter relative to summer and fall, with clusters in fatty acid beta-oxidation, the electron transport chain, and protein targeting to the mitochondria (Figure 5). These proteome changes correspond well with changes in most other tissues of hibernators, principally denoting the shift to lipid metabolism during hibernation as animals rely on fat stores for energy. As platelets appear to be no exception, it is conceivable that they hold similar cell autonomous protection from cooling damage as found in hamster and ground squirrel smooth muscle cells, kidney cells and neurons. Interestingly, ground squirrel neurons maintain their tubulin network during cooling by limitation of the production of reactive oxygen species (Ou et al., 2018). Such mechanism may also support tubulin stability and possibly underlie the difference in cooling induced platelet shape change between hibernators (reversible rod formation) and non-hibernators (irreversible sphere formation). Given this clearcut difference in response to cooling and the ease of obtaining primary cells, platelets constitute an important platform for the future study of cell-autonomous adaptations in hibernators.

**FIGURE 6:**
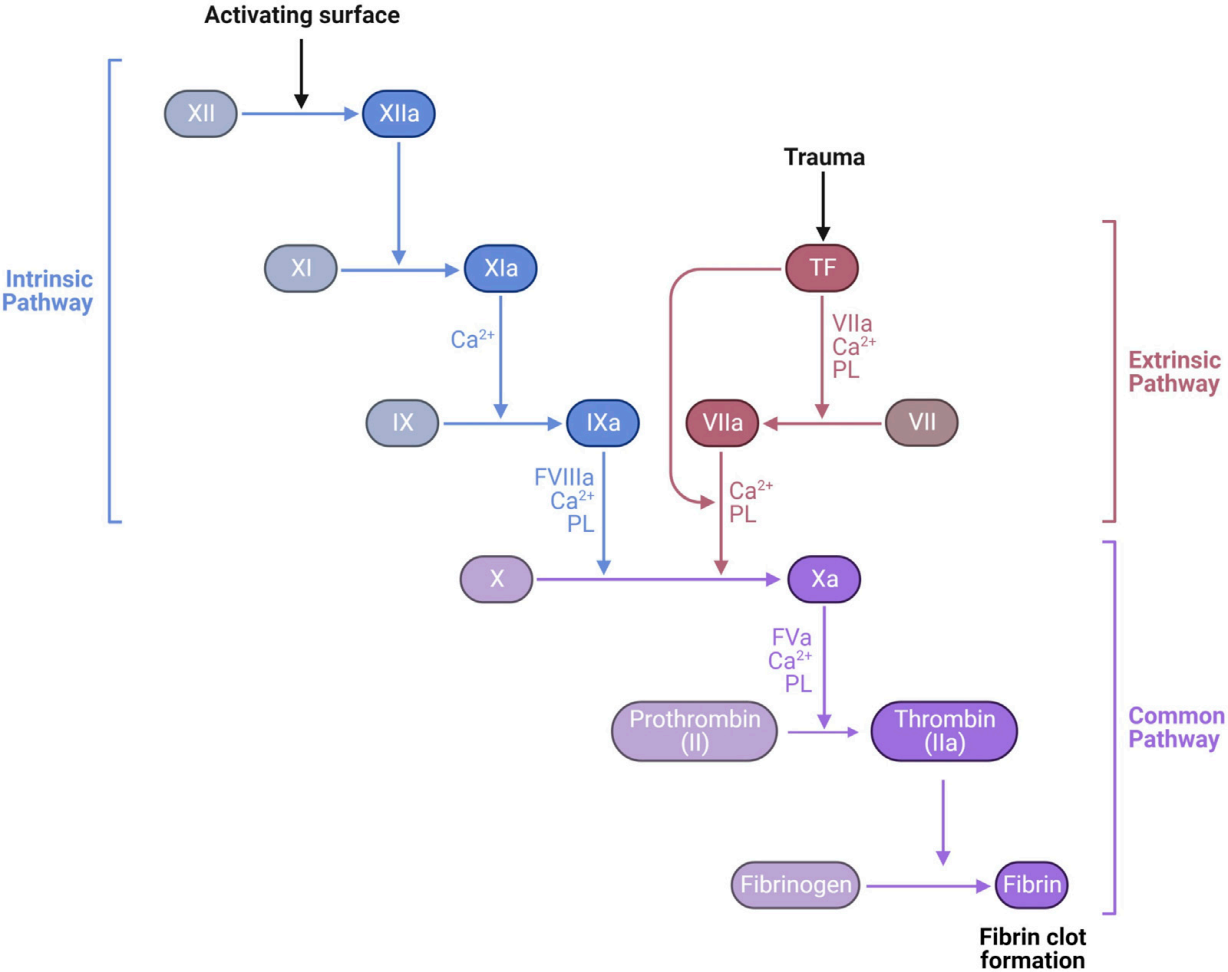
Laboratory measurement of the coagulation cascade is divided into the intrinsic pathway, measured by activated partial thromboplastin time (APTT) dependent on factors XII, XI, IX, and VIII, and the extrinsic pathway, measured by prothrombin time (PT) dependent on factor VII. Both APTT and PT also depend on the common pathway factors II (prothrombin), V, X, and fibrinogen. The final product of the common pathway is activation of thrombin (IIa) which cleaves fibrinogen (I) into fibrin (1a) forming a clot https://biorender.com/.

Secondary hemostasis occurs simultaneously with primary hemostasis forming a fibrin network to trap red and white blood cells and further strengthen the hemostatic platelet plug (Figure 6). Secondary hemostasis occurs via a plasma coagulation cascade divided into the intrinsic and extrinsic pathways. Most clotting factors are serine proteases that are always present in plasma as inactive zymogens (identified by Roman numerals II, VII, IX, X, XI, and XII) and are activated by proteolysis (addition of an “a”). Factors V and VIII are non-enzymatic cofactors also activated by proteolysis. The extrinsic pathway starts with exposure and binding of tissue factor (TF) to plasma coagulation factor VII, which forms a TF/VIIa complex. The TF/VIIa complex proteolytically activates factors IX and X, commencing the common pathway, creating a prothrombinase complex with Va that converts prothrombin (factor II) into thrombin (IIa) (Versteeg et al., 2013). Thrombin slowly accumulates during the amplification phase, activating platelets and platelet derived factor V, amplifying the prothrombinase activity. In a positive feedback loop, thrombin activates factor XI and VIII, generating more factor Xa. Hemophilia A and B are defined by deficiencies in factors VIII and IX, respectively, which is associated with spontaneous and prolonged bleeding. The extrinsic pathway can be assayed *in vitro* by measuring the prothrombin time (PT). The intrinsic pathway of coagulation can be triggered independently by collagen, polyphosphates secreted by platelets, neutrophil extracellular traps (NETs), and artificial material such as glass, leading to activation of factors XII, XI, kallikrein and the subsequent downstream coagulation factors (Muller et al., 2009; Versteeg et al., 2013). The intrinsic pathway can be assessed *in vitro* by measuring the activated partial thromboplastin time (APTT).

### Secondary hemostasis adaptations during torpor

**FIGURE 7:**
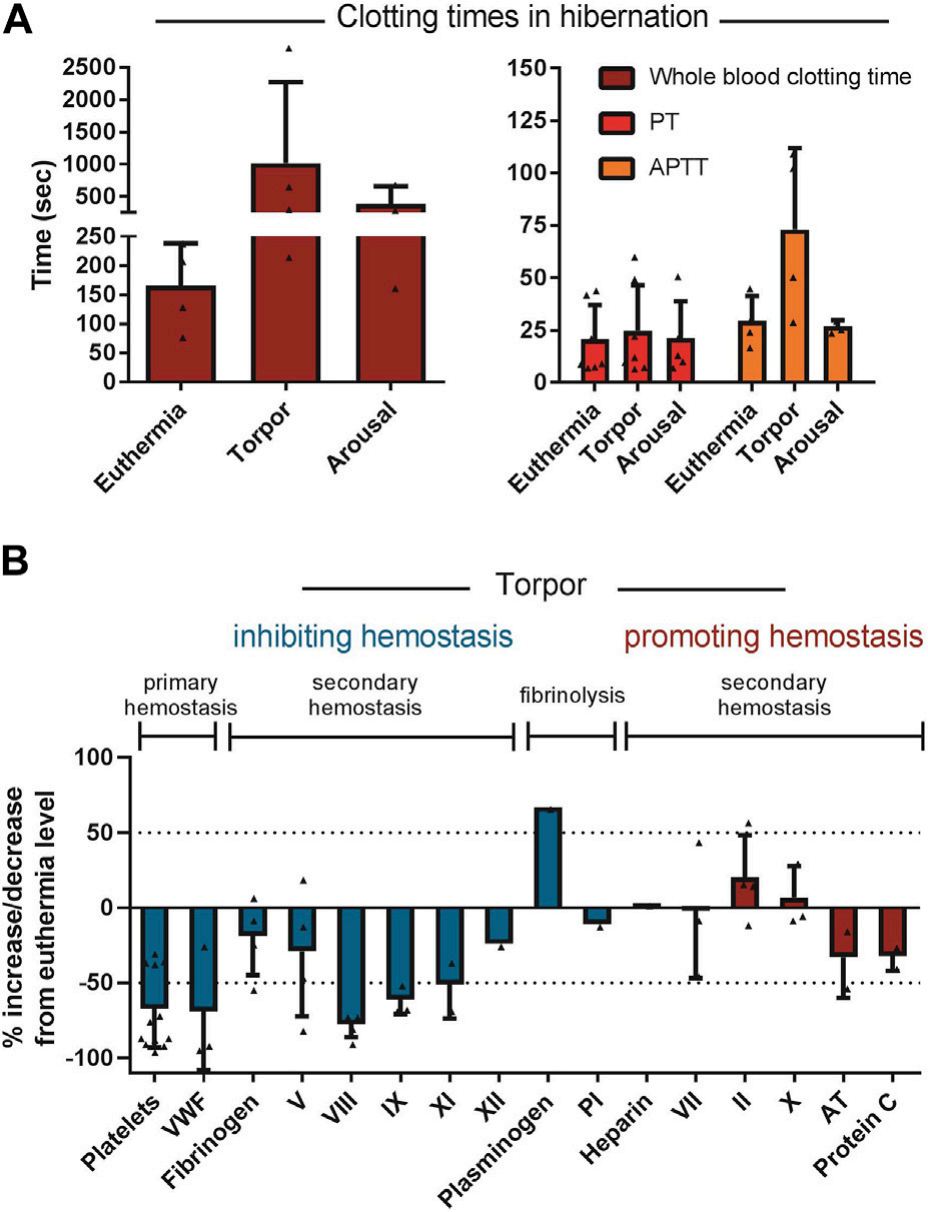
Regulation of components of primary hemostasis, secondary hemostasis, and fibrinolysis tilts towards inhibition of hemostasis during torpor. **(A)** Whole blood clotting time and APTT are prolonged during torpor in several species and return to normal during arousal. In contrast, PT is not consistently prolonged. **(B)** All factor levels are normalized as percentage of euthermia level. If a study did not report euthermic level, literature data was used. Data is represented as mean and standard deviation, with each triangle representing the data from an individual study listed in Table 2.

Secondary hemostasis is reduced during torpor in ground squirrels, bears, hedgehogs, and hamsters. In general, hibernating animals in torpor reduce the level of coagulation factors VIII, IX and XI resembling mild hemophilia A, B and C respectively (Denyes and Carter, 1961; Biorck et al., 1962; Lechler and Penick, 1963; Cooper et al., 2016; Welinder et al., 2016). The decreases in factor VIII, IX and XI during torpor in examined hibernating species are on average 78%, 61% and 51%, respectively, compared to euthermic non-hibernating values (Figure 7). Interestingly, the extrinsic pathway of the coagulation cascade remains fairly unaltered during hibernation. A logical explanation could be that thrombosis due to stasis of blood should be prevented, but blood clotting due to tissue damage by an intruder or predator (activating the extrinsic pathway) may still be vital. An alternative explanation is that during torpor, damage to blood vessels is unlikely, so suppressing the extrinsic pathway is not necessary and protection against stasis-induced clots is more important. Remarkably, one clotting factor consistently showed an increase in protein levels during torpor: prothrombin. Possibly, this is caused by a decrease in its normal baseline formation resulting from inhibition of spontaneous, low-level activation of the clotting cascade, thus diminishing the conversion of prothrombin into thrombin (Lechler and Penick, 1963; Welinder et al., 2016). Prothrombin mRNA levels were slightly decreased in the livers of hibernating 13-lined ground squirrels, consistent with this explanation (Gillen et al., 2021). To assess the overall level of activation of clotting, levels of the irreversible complex between thrombin and its inhibitor antithrombin may be used. Thrombin-antithrombin complexes were decreased during hibernation in 13-lined ground squirrels, consistent with suppression of secondary hemostasis (Bonis et al., 2019). An alternative to reducing clotting factors would be to increase anticoagulants. Yet, the anticoagulant proteins antithrombin and protein C are not reduced in torpid hamsters and ground squirrels (Bonis et al., 2019). However, in hibernating bears antithrombin levels are reduced, but it is not known if this is due to decreased production of antithrombin or increased consumption (Welinder et al., 2016). Collectively, torpor features the suppression of both the intrinsic and extrinsic arms of the coagulation cascade leading to prolonged APTT times and a mild extension of PT times (Biorck et al., 1962; Lechler and Penick, 1963; Pivorun and Sinnamon, 1981; De Wit et al., 1985; Iles et al., 2017). Diverse hibernators including shallow hibernators (bears), facultative hibernators (hamsters), and deep seasonal hibernators (ground squirrels and bats) use similar adaptations. These common strategies reduce both primary and secondary hemostasis leading to prolonged APTT (Figure 7).

**TABLE 2:** Secondary hemostasis in hibernation.

Measurement	Euthermia (EU)	Torpor	Arousal	Species	References
Thrombin time (sec)	13.2 ± 0.7	15.8 ± 0.7*	14.7 ± 1.3	Franklin’s ground squirrel	Pivorun and Sinnamon (1981)
	37°C	6°C	37°C		
PT (sec)	9.8 ± 1.6	10.6 ± 1.5		13-lined ground squirrel	Lechler and Penick (1963)
	36.3°C	7.9°C			
	8.1 ± 0.3	8.3 ± 0.2	14.0 ± 2.1 *^,#^	Franklin’s ground squirrel	Pivorun and Sinnamon (1981)
	37°C	6°C	37°C		
	42.6	60.5	51.2	European Hedgehog	De Wit et al. (1985)
	8.75 ± 0.5			Golden hamster	Deveci et al. (2001)
	41–48	38–62		Red-eared slider (turtle); Painted turtle	Barone and Jacques (1975)
	8.6 ± 0.3	7.6 ± 1.5	8.1 ± 2.2	American black bear	Iles et al. (2017)
	22	23	22	Hedgehog	Biorck et al. (1962)
(%EU)	100% ± 3% (n = 5) 37°C	129% ± 16% * (n = 5) 20°C	110% ± 4%^#^ (n = 5) 35°C	C57Bl/6 pharmacological torpor	(De Vrij et al. unpublished)
(%EU)	100% ± 9% (n = 10)	184% ± 82% (n = 3)	84% ± 6%^#^ (n = 4)	Syrian hamster	(De Vrij et al. unpublished)
	36°C	9°C	37°C		
APTT (sec)	45.5 ± 8.7	109.3 ± 42*		13-lined ground squirrel	Lechler and Penick (1963)
	36°C	8°C			
	25.0 ± 1.0	51.0 ± 2.4*	30.1 ± 1.4^#^	Franklin’s ground squirrel	Pivorun and Sinnamon (1981)
	36°C	6°C	37°C		
	29.8 ± 0.27			Golden hamster	Deveci et al. (2001)
	17.6 ± 0.8	29.7 ± 7.9*	24.5 ± 2.1*	American black bear	Iles et al. (2017)
(%EU)	100% ± 22% (n = 10)	339% ± 88% (n = 3)	86% ± 12%^#^ (n = 4)	Syrian hamster	(De Vrij et al. unpublished)
	36°C	9°C	37°C		
Thrombin generation (%EU)	100% ± 23% (n = 3) 36°C	8% ± 19%* (n = 7) 9°C	61% ± 58% (n = 5) 37°C	Syrian hamster	(De Vrij et al. unpublished)
Prothrombin (U/mL)	443 ± 132 36.3°C	698 ± 143* 7.9°C		13-lined ground squirrel	Lechler and Penick (1963)
(IU/mL)	1.10 ± 0.19	1.29 ± 0.31*		Scandinavian brown bear	Welinder et al. (2016)
(Sec)	11.5 ± 0.3 37°C	11.9 ± 0.3 6°C	10.5 ± 0.3*^,#^ 37°C	Franklin’s ground squirrel	Pivorun and Sinnamon (1981)
(U/mL)	20	55	90	Hedgehog	Biorck et al. (1962)
(%EU)	100% ± 10% 36°C	116% ± 20% 9°C	184% ± 15% * 37°C	Syrian hamster	(De Vrij et al. unpublished)
Residual prothrombin in serum (U/mL)	27 ± 38 36°C	449 ± 157* 8°C		13-lined ground squirrel	Lechler and Penick (1963)
Factor II, VII, X combined assay (sec)	12.8 ± 0.4 37°C	14.3 ± 0.6 6°C		Franklin’s ground squirrel	Pivorun and Sinnamon (1981)
Factor V	639% ± 212% 36°C	570% ± 143%		13-lined ground squirrel	Lechler and Penick (1963)
		7.9°C			
(Sec)	16.2 ± 0.3	20.1 ± 0.3*	17.8 ± 0.4*^,#^ 37°C	Franklin’s ground squirrel	Pivorun and Sinnamon (1981)
	37°C	6°C			
(% human plasma)	450	542	354	Hedgehog	Biorck et al. (1962)
(%EU)	100% ± 44%	20% ± 5% *	72% ± 26%	Syrian hamster	(De Vrij et al. unpublished)
	36°C	9°C	37°C		
Factor VII	369% ± 138% 36°C	536%		13-lined ground squirrel	Lechler and Penick (1963)
		8°C			
	1.01 ± 0.6	0.57 ± 0.14		Scandinavian brown bear	Welinder et al. (2016)
(%EU)	100% ± 15%	93% ± 22%	133% ± 11%	Syrian hamster	(De Vrij et al. unpublished)
	36°C	9°C	37°C		
Factor VIII	165% ± 73% 36°C	35% ± 11%*		13-lined ground squirrel	Lechler and Penick (1963)
		8°C			
(% relative to human plasma)	232 ± 2.0	68 ± 0.1*	230%	13-lined ground squirrel	Cooper et al. (2016)
(IU/mL)	2.92 ± 1.03	0.86 ± 0.35 *		Scandinavian brown bear	Welinder et al. (2016)
(%EU)	100% ± 15%	11% ± 5% *	52% ± 37%	Syrian hamster	(De Vrij et al. unpublished)
	36°C	9°C	37°C		
Factor IX (% human plasma)	378 ± 157	188 ± 65*		13-lined ground squirrel	Lechler and Penick (1963)
	36°C	8°C			
	425 ± 20	140 ± 4.0*	380%	13-lined ground squirrel	Cooper et al. (2016)
(%EU)	100% ± 13%	34% ± 10%	150% ± 23%^#^	Syrian hamster	(De Vrij et al. unpublished)
	36°C	9°C	37°C		
Factor X	867% ± 126% 36°C	805% ± 269% 8°C		13-lined ground squirrel	Lechler and Penick (1963)
(Sec)	18.9 ± 0.4	18.7 ± 0.7	19.4 ± 0.3	Franklin’s ground squirrel	Pivorun and Sinnamon (1981)
	37°C	6°C	37°C		
(%EU)	100% ± 16%	131% ± 30%	138% ± 13%	Syrian hamster	(De Vrij et al. unpublished)
	36°C	9°C	37°C		
Factor XI (%)	111	72		13-lined ground squirrel	Lechler and Penick (1963)
	36°C	8°C			
(%EU)	100% ± 22%	34% ± 14% *	92% ± 31%^#^	Syrian hamster	(De Vrij et al. unpublished)
	36°C	9°C	37°C		
Factor XII	291% ± 71% 36°C	222% ± 70%		13-lined ground squirrel	Lechler and Penick (1963)
		8°C			
Fibrinogen (mg%)	189 ± 49	145 ± 34		13-lined ground squirrel	Lechler and Penick (1963)
	36°C	8°C			
(g/L)	2.09 ± 0.94	2.26 ± 0.46		Scandinavian brown bear	Welinder et al. (2016)
(%)	0.54	0.5	0.29	Hedgehog	Biorck et al. (1962)
(%EU)	100% ± 20%	45% ± 20%	130% ± 65%^#^	Syrian hamster	(De Vrij et al. unpublished)
	36°C	9°C	37°C		
Plasminogen (%EU)	100% ± 31% (n = 10)	168% ± 51% (n = 5)	249% ± 51% (n = 5) *		(De Vrij et al. unpublished)
(% relative to human plasma)	37.6% ± 9.2%	19.7% ± 12.3%		13-lined ground squirrel	Bonis et al. (2019)
PAI-1 (ng/mL)	2.15 ± 1.04	0.45 ± 0.35		13-lined ground squirrel	Bonis et al. (2019)
Plasmin inhibitor (%EU)	100% ± 5%	89% ± 18%	109% ± 24%	Syrian hamster	(De Vrij et al. unpublished)
	36°C	9°C	37°C		
Protein C (IU/mL)	0.44 ± 0.08	0.33 ± 0.08*		Scandinavian brown bear	Welinder et al. (2016)
(%EU)	100% ± 12%	61% ± 9%	112% ± 14%^#^	Syrian hamster	(De Vrij et al. unpublished)
	36°C	9°C	37°C		
Antithrombin (IU/mL)	0.98 ± 0.09	0.47 ± 0.04*		Scandinavian brown bear	Welinder et al. (2016)
(%EU)	100% ± 6%	86% ± 11%	104% ± 7.2%	Syrian hamster	(De Vrij et al. unpublished)
	36°C	9°C	37°C		
Heparin (sec)	36.6 ± 0.3 37°C	37.7 ± 0.4		Franklin’s ground squirrel	Pivorun and Sinnamon (1981)
		6°C			
D-dimer (ug/L)		152	124	American black bear	Iles et al. (2017)
	15 ± 13	28 ± 32	10 ± 17	Syrian hamster	de Vrij et al. (2021)
	36.3°C ± 0.9°C	8.8°C ± 0.7°C	36.7°C ± 1.1°C		
(%EU)	100% ± 55%	76% ± 22%	80% ± 26%	13-lined ground squirrel	Bonis et al. (2019)

The decreased activities of clotting factors in the blood correlate well with the changes in APTT and PT, however, the differences in protein levels could be due to decreased synthesis or increased consumption during torpor. Many of the blood proteins involved in secondary hemostasis are produced in liver, and a few are released from platelets and endothelial cells. In addition to traditional activity assays, transcriptome, proteome, and metabolomes are being analyzed in liver and plasma to identify changes in hemostatic regulators. The livers of torpid 13-lined ground squirrels revealed decreases in prothrombin, factor V, factor IX, tissue factor, and heparin cofactor II mRNA while VWF mRNA was decreased in bone marrow (Cooper et al., 2016; Gillen et al., 2021). There was also an increase in α2 macroglobulin mRNA in liver during hibernation, a serine protease inhibitor that can inhibit a broad range of clotting factors and its protein level has been previously shown to increase during torpor (Srere et al., 1995), potentially contributing to the suppressed state of hemostasis in torpor. Although these findings are in line with a suppression of hemostasis in torpor, some other studies demonstrate changes that are less unidirectional or involve both procoagulant and anticoagulant factors. For example, clotting factor mRNA expression was unexpectedly decreased in the brains of hibernating Himalayan marmots, but not in their livers (Bai et al., 2019). Another marmot transcriptome study compared two species, but did not look into seasonal variation in expression (Liu et al., 2016). While transcriptomes are useful in measuring new synthesis of proteins, activity in plasma is ultimately estimated by measuring levels of circulating protein which can be detected using proteomics. The bear plasma proteome revealed decreases in clotting factors similar to that reported for individual factor assays with winter increases in α2 macroglobulin, potentially inhibiting thrombin, whereas thrombin itself and fibrinogen were also increased. In contrast, factors VIII and IX and VWF decreased in winter, in line with a suppression of hemostasis, but also the anticoagulant factors antithrombin and protein C decreased (Welinder et al., 2016). An arctic ground squirrel liver proteome also revealed increased α2 macroglobulin but a decrease in antithrombin during torpor (Shao et al., 2010). Finally, a liver proteome of 13-lined ground squirrels showed no seasonal differential expression of proteins involved in hemostasis (Rose et al., 2011). Although all analyzed hibernating mammals demonstrated some suppression within the hemostatic system, there remains some variation between species regarding which factors are involved and whether some procoagulant factors are increased. For the most part proteomic studies seem to align better with assays of individual proteins than do transcriptomic studies. This suggests that protein levels may be regulated more by consumption than transcriptionally. Further analysis of the plasma and liver proteomes may reveal other adaptations that regulate hemostasis.

### Fibrinolysis adaptations during torpor

Proteins released from endothelial cells after clot formation initiate fibrinolysis. The cross-linked fibrin network is enzymatically degraded by plasmin, which is formed from plasminogen by tissue plasminogen activator (t-PA). t-PA is slowly released by damaged endothelium enabling a gradual degradation of fibrin after the bleeding has stopped and tissue regeneration has started (Gonias, 2021). A marker of fibrinolysis activation is the level of circulating complexes between tPA and its inhibitor plasminogen activator inhibitor 1 (PAI-1). In ground squirrel torpor, PAI-1 levels are decreased along with tPA-PAI-1 complexes consistent with a hyperfibrinolytic state (Bonis et al., 2019). In ground squirrels, plasminogen levels drop two-fold while in bears and hamsters an increase is seen during torpor. Upon fibrinolysis, fibrin in a clot is cleaved into fibrin degradation products, of which D-dimers can be detected in plasma and is commonly used in the diagnosis of venous or arterial thrombosis. In bears, hamsters, and ground squirrels no increases in D-dimers are seen during hibernation, consistent with low levels of clot formation and fibrinolysis (Iles et al., 2017; Bonis et al., 2019). The lack of D-dimers is consistent with suppression of secondary hemostasis and absence of actual clot fibrinolysis during torpor, while the hyperfibrinolytic state reflected by increased tPA and PAI-1 may be an added layer of protection in the event that a clot does form (Figure 7).

## Medical applications

The pathways that prevent activation and clearance of circulating platelets throughout hibernation, key receptors and ligands for platelet margination, and suppression of activation and apoptosis in the cold remain to be determined. Yet, unraveling these pathways could have direct applications in the storage of human platelets. Mimicking the hibernator’s platelet resilience to extended cold storage by unlocking their cell-autonomous adaptations might allow long-term cold storage for human platelet transfusion, thus improving transfusion availability and reducing costs. Human red blood cells can be stored in the cold, and research on arctic ground squirrel red blood cells stored in the cold revealed novel protective mechanisms that may be applicable to human red blood cells, but is beyond the scope of this review on hemostasis and hibernation (Gehrke et al., 2019). Additionally, expanding our knowledge about the effects of lower temperature on hemostasis may help understand its role in patients with accidental hypothermia, e.g., near drowning, or those undergoing therapeutic hypothermia. Advancement of these insights will help physicians to better evaluate advantages and disadvantages of therapeutic hypothermia. More applications for therapeutic hypothermia may arise, as mild intraoperative hypothermia may for instance be beneficial in cardiac surgery (Hendriks et al., 2022) and plastic surgery by reducing thrombosis in free tissue transfer, hence improving free flap survival (Liu et al., 2011).

Understanding the mechanisms that hibernators use to prevent activation and clearance of platelets, and prevent thromboembolic complications, may be relevant for other medical applications other than surgery. Pulmonary embolism arising from DVT is a serious medical condition caused by immobilization leading to activation of blood clotting. A study in bears revealed the role of decreased platelet HSP47 in preventing clot formation (Thienel et al., 2023), and this could be translated to humans and other mammals under immobilization. This research could have direct applications in screening patients at risk of DVT with elevated HSP47 and also by developing therapeutic treatments to block HSP47 in immobilized patients. Diffuse intravascular coagulation (DIC) is a severe complication that can arise in critically ill patients. Sepsis, a syndrome of organ failure due to a dysregulated host response to infection, is a common cause of critical illness and DIC. DIC is associated with thromboembolic complications on the one hand and bleeding complications due to consumption of platelets and coagulation factors on the other hand. Therapeutic hypothermia may improve the coagulopathy as measured by thromboelastography in patients with sepsis or septic shock (Johansen et al., 2015), and even decrease mortality and end-organ damage in sepsis as demonstrated experimentally (Acosta-Lara and Varon, 2013). Preventing fever and maintaining normothermia (37°C) by cooling volunteers upon injection of endotoxin, decreases markers of DIC that were induced by endotoxin challenge (Harmon et al., 2021). Yet, therapeutic hypothermia did not lower mortality in sepsis (Itenov et al., 2018).

The use of anticoagulant drugs to prevent thromboembolic complications in high-risk situations after major surgery, in patients with atrial fibrillation, or after a previous unproven thromboembolic event, is associated with bleeding complications as major side effect. Moreover, bleeding is the most common adverse drug event bringing patients to emergency wards (Leendertse et al., 2008; Budnitz et al., 2011) and the newest class of anticoagulants, direct oral anticoagulants (DOAC), are associated with fatal bleeding on 0.16 per 100 patient-years (Chai-Adisaksopha et al., 2015). Therefore bleeding requires correction of anticoagulation as quickly as possible. If a pharmacological tool becomes available to mimic the torpor induced suppression of thrombosis, it may add the benefit of a rapid reversal strategy, since arousal rapidly reverses antithrombotic effects within minutes to hours. However, most reversal techniques require several hours to reverse anticoagulation, i.e., by administering fresh frozen plasma, Vitamin K or prothrombin complex concentrate (Fredriksson et al., 1992; Hanley, 2004). To date, only two registered monoclonal antibody fragments against DOAC are faster and reverse anticoagulation within minutes (White et al., 2022), but are highly expensive. Therefore, the development of new treatment and reversal strategies may help physicians to effectively manage the life-threatening emergency of bleeding in anticoagulated patients (Christos and Naples, 2016). Unraveling the mechanisms driving the hemostatic adaptations in hibernators might contribute to the development of these novel, reversible and safer anticoagulants with lower risk on coagulation abnormalities—even at euthermia—in patients with critical illnesses like sepsis, trauma and after major surgery.

## Summary and future research

**FIGURE 8:**
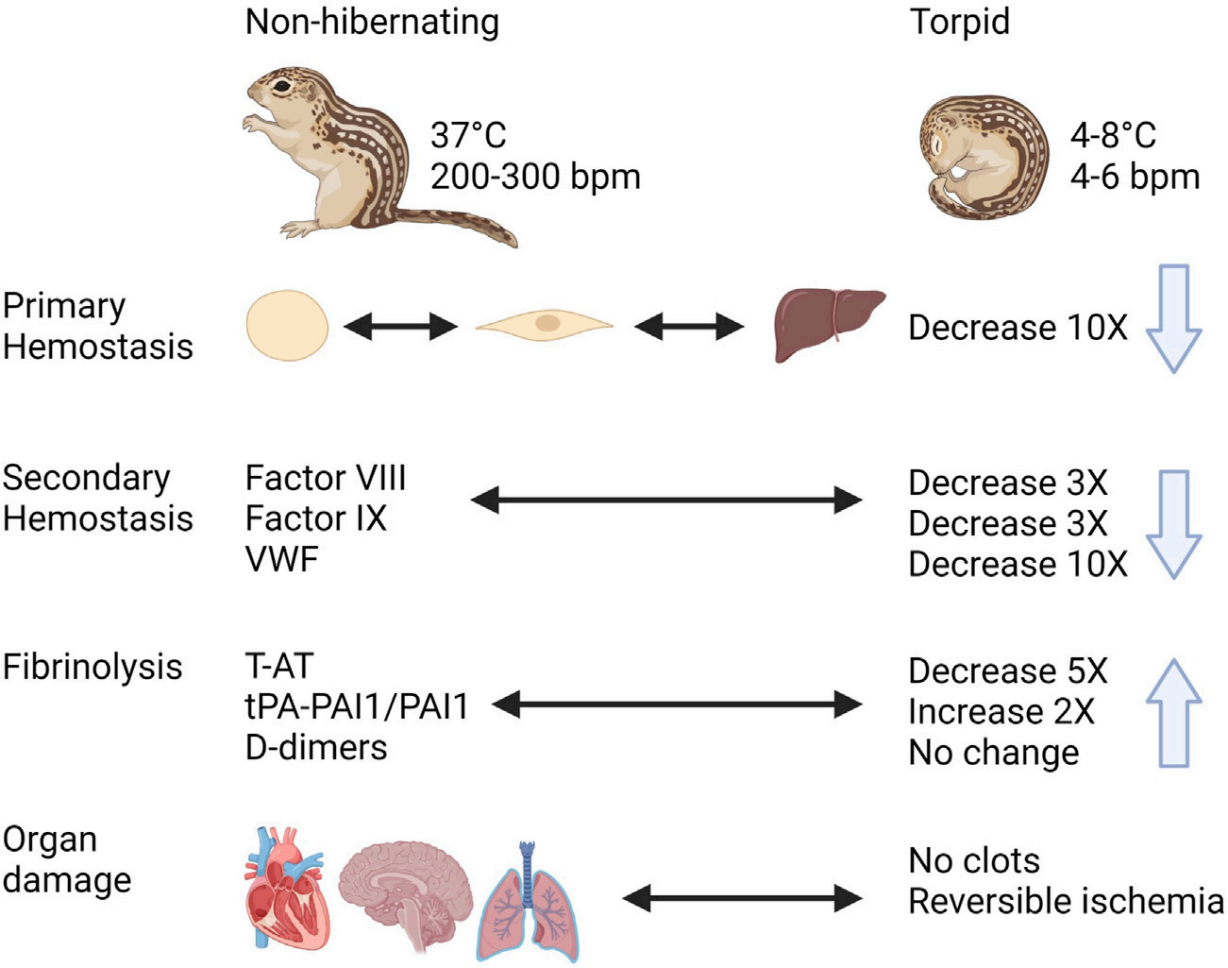
Graphical summary of trends in regulation of hemostasis in a hibernating mammal https://biorender.com/.

In spite of the expected risk of thrombosis during hibernation due to prolonged immobility with reduced blood flow and increased blood viscosity, hibernating mammals do not show signs of thrombotic complications during or after hibernation (Figure 8). During torpor in diverse hibernating species of mammals, there is a rapid and reversible anti-thrombotic shift by the reduction in circulating platelets, VWF, and coagulation factors. This results in a mixed phenotype resembling thrombocytopenia, Von Willebrand Disease, and Hemophilia A, B and C. At the same time, torpid animals maintain factors required for fibrinolysis. One of the most remarkable adaptations is the ability to store platelets in the liver sinusoids during torpor, releasing them intact after prolonged periods of storage in the cold. Future research should use phosphoproteome and metabolomics studies to identify seasonal differences in signaling pathways and molecules released by platelets, in addition to characterizing mitochondrial function. Many common adaptations protect hibernating mammals from unwanted activation of hemostasis during torpor and yet allow for a quick reversal of this state to restore proper hemostasis during arousal. The mechanisms behind these reversible adaptations remain to be discovered and may open doors for novel therapies and improved treatments.
